# Evaluation of children with urolithiasis

**DOI:** 10.4103/0970-1591.36717

**Published:** 2007

**Authors:** Syed A.H. Rizvi, Sajid Sultan, Mirza N. Zafar, Bashir Ahmed, Syed M. Faiq, Kehkashan Z. Hossain, Syed A.A. Naqvi

**Affiliations:** Department of Urology, Sindh Institution of Urology and Transplantation, Civil Hospital, Karachi - 74200, Pakistan; * Department of Pathology, Sindh Institution of Urology and Transplantation, Civil Hospital, Karachi - 74200, Pakistan; ** Department of Radiology, Sindh Institution of Urology and Transplantation, Civil Hospital, Karachi - 74200, Pakistan; *** Department of Nutrition, Sindh Institution of Urology and Transplantation, Civil Hospital, Karachi - 74200, Pakistan

**Keywords:** Evaluation, pediatric, urolithiasis

## Abstract

**Objectives:**

To describe an evaluation protocol for pediatric stone formers for risk assessment and management strategies.

**Materials and Methods:**

Between 2002-2006, 2618 children of age three months to 15 years were evaluated for stone disease. Evaluation included demographics, history, anthropometry, diet, ultrasound, X-ray KUB, IVU, blood and 24h urine chemistry and cultures. Stones were analyzed by IR spectroscopy.

**Results:**

The median age was seven years with a M:F ratio of 2.2:1. Of the 2618 patients, 2216 presented with normal renal function and 402 with renal failure. Main symptoms were abdominal pain (33%), flank pain (38%) and fever (38%). Renal failure patients also had shortness of breath (38%) and oligo-anuria (26%). Children were malnourished with height and weight deficits in 65% and 76% respectively. Diet was low in protein (74%), calcium (55%) and fluids in (55%), high in oxalate (55%), sodium (39%), purines (42%) and refined sugar (41%). Overall urine cultures were positive in 1208 (46%) with E. coli (38%) and Klebsiella (8%).

Stone distribution was renal in 64%, ureter in 8%, bladder in 18%, bilateral in 40% and multiple sites in 18%. Median stone size was >1.5-2.0 cm. The frequency of compounds in stones was ammonium urate (58%), calcium oxalate (63%), uric acid (6%), calcium phosphate (12%) and struvite (8%). Metabolic abnormalities included hypovolumia (31%), hypocitraturia (87%), hyperoxaluria (43%) and hyperuricosuria (26%). Dietary and medical treatment corrected risk factors in two-thirds of patients with a recurrence rate of about 1.15%.

**Conclusion:**

An evaluation based on history, imaging, diet, metabolic analysis and stone type can help to tailor management strategies.

Pakistan lies in the middle of the Afro-Asian stone belt stretching from Sudan and Egypt in the West to Philippines in the East, geographically falling within the tropical and subtropical regions. Historically, the first survey of stone disease in the subcontinent was undertaken by Col. McCarrison of the Indian Medical Service in 1931 where the highest incidence was found in Sindh and Punjab provinces which now constitute Pakistan.[1] Recent studies also show a high prevalence of urinary stones both in the adult and pediatric populations.[2] However, in recent years a change of pattern has been observed from predominantly bladder calculi in the 1980s constituting 76% of all stones to predominantly renal calculi in 85% of the patients in the present decade. An unfortunate aspect of stone disease in Pakistan is neglect and delay in seeking treatment which results in many children presenting with renal failure.[3]

On this backdrop, evaluation of children with stone disease becomes of paramount importance both in terms of management and development of preventative strategies. This paper describes our approach to evaluate children with stone disease so as to develop a meaningful diagnostic protocol for developing countries. This can help in the management and prevention of stone recurrence in the pediatric population.

## MATERIALS AND METHODS

Between January 2002 and December 2006, 2618 pediatric patients of age three months to 15 years, presented with urolithiasis in the outpatient department (OPD) and Emergency room (ER) of a tertiary care hospital. Of these 2618 patients, 2216 (85%) had normal renal functions and 402 (15%) had associated renal failure. In the renal failure group, 105 (26%) were oligo-anuric and 297 (74%) were non-anuric.

**Figure 1:**
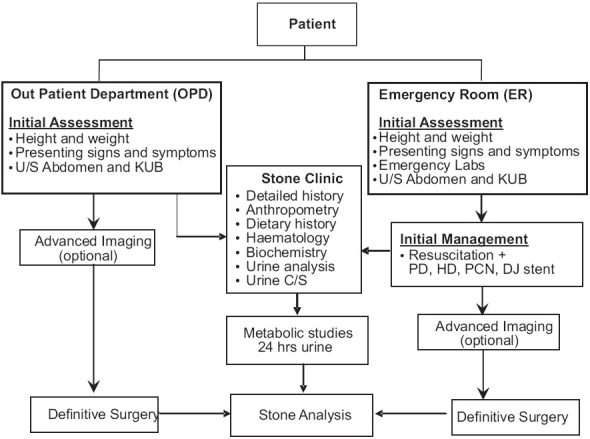
Evaluation protocol of pediatric stone formers

All patients presenting to the OPD or ER were evaluated according to the protocol listed in [Figure 1]. All information was collected and documented according to the two separate preset proforma for stone patients with and without renal failure.

Depending on the associated clinical conditions i.e. renal failure, advanced uremia, urosepsis, the following investigations were undertaken on case to case basis. Arterial blood gases (ABGs), culture of blood, serology for Hepatitis B and C, clotting profile and X-ray chest. These patients required resuscitation and early intensive management including dialysis and/or relief of obstruction as initial management.

### Detailed evaluation of stone patients included:

Demographic: Age, sex, family history, socioeconomic status, address of residence including rural/urban location.

1. Anthropometric analyses were evaluated by guidelines of National Centre for Health Statistic (NCHS)[4] by height, weight and mid-arm circumference and triceps skin fold thickness.
2. Dietary analysis: Evaluation of diet was undertaken by a food frequency questionnaire and a seven-day dietary recall proforma. The food frequency evaluated intake of foods associated with urolithiasis. These included animal protein, calcium, oxalate, phosphates, purine, sodium, potassium and refined sugar.[5] Twenty-four-hour fluid intake was assessed by number of glasses consumed (one glass=250 ml). Criteria for normal fluid intake for children < 10 kg weight was 150 ml/kg for first 5 kg and 50 ml/kg for the second 5 kg. For children > 10 kg weight, 100 ml/kg for first 10 kg, 50 ml/kg for second 10 kg and 20 ml/kg thereafter.
3. Radiology and imaging evaluation: Ultrasound abdomen including kidney, ureter and bladder (KUB) was undertaken in all cases. Intravenous urogram (IVU) was done in patients with normal renal functions requiring extracorporeal shockwave lithotripsy (ESWL) or percutaneous nephrolithotomy (PCNL). A CT scan was undertaken only as and when required. Radio-isotope scans (nuclear imaging) were used to assess the relative renal function in compromised kidneys.
4. Detailed metabolic evaluation included serum calcium, phosphorus, uric acid, magnesium and proteins. Twenty-four-hour urine collection was done with thymol preservation. Urine collection required catheterization for 24h in small children who were not continent. Tests included creatinine, calcium, uric acid, sodium, potassium, magnesium, ammonium, citrate, oxalate and protein. Cystine was checked qualitatively by sodium nitropruside solution. Adequacy of collection was checked by the formula, Urine creatinine correction = urinary creatinine mg/dl × 24h urine volume ÷ 100 × body weight. Reference ranges for blood and urine chemistry were adopted from Textbook of Pediatrics[6] and Hesse,[7] where values were standardized by reporting results in mg/kg/24h [Table 1].
5. Stone analysis was done by infrared spectroscopy and by florescent infrared spectroscopy (FTIR). Whenever possible separate analysis was done for core, middle and surface of each stone. Chemical examination was undertaken in stones with mixed compounds.
6. Management: UTI was treated according to sensitivity pattern in both groups of patients, with and without renal failure. Definitive management included open surgery for large stone burden in the urinary tract, kidneys with reduced cortex and stones with UTI and structural abnormalities.

**Table 1:** Normal values for serum and 24h urine chemistry

Blood chemistry	Normal ranges	24h urine biochemistry	up to age 9	>9 – 15
S. Urea (mg/dl)	12–45	Urine volume (ml/24h)	500 – 1000	700 – 1400
S. Creatinine (mg/dl)	0.3 – 1.0	Urinary ammonium (mmol/24h)	20 – 50	20 – 50
S. Uric Acid (mg/dl)		Uric Acid (mg/kg/24h)	10 – 15	10 – 15
1 – 5 years	1.7 – 5.8	Calcium (mg/kg/24h)	1.5 – 4.5	1.5 – 4.0
6 – 11 years	2.2 – 6.6	Phosphorus (mg/kg/24h)	8 – 18	8 – 18
< 12 years	3.0 – 7.7	Protein (mg/24h)	< 150	< 150
S. Calcium (mg/dl)	8.8 – 10.8	Sodium (mEq/24h)	40 – 90	60 – 175
S. Phosphorus (mg/dl)	4.0 – 7.0	Potassium (mEq/24h)	10 – 50	20 – 75
S. Magnesium (mg/dl)	1.5 – 3.1	Oxalate (mg/kg/24h)	0.3 – 1.0	0.5 – 1.5
S. Sodium (mEq/L)		Citrate (mg/kg/24h)	5 – 8	7 – 15
Up to 12 years	138 – 145	Magnesium (mg/kg/24h)	1.2 – 2.5	1.0 – 1.5
> 12 years	136 – 146	pH	6 – 7	6 – 7
S. Potassium (mEq/L)	3.5 – 5.0		
Chloride (mEq/L)	98 – 107			
Bicarbonate (mEq/L)	20 – 28			
T. Protein (gm/dl)				
1 – 7 years	6.1 – 7.9			
8 – 12 years	6.4 – 8.1			
13 – 19 years	6.6 – 8.2			
S. Albumin (gm/dl)	3.5 – 5.0			

Minimally invasive treatment, PCNL, ureterorenoscopy (URS), cystolithoclast and ESWL were performed in those with small burden, good renal cortex and without UTI.

Dietary and pharmacologic management was undertaken according to identified risk factors. These included dietary intervention, alkalization of urine, allopurinol, pyridoxine and thiazides where indicated. Patients were followed up in a dedicated pediatric stone clinic with repeat metabolic workup between three and 12 months after stone-free status.

### Statistical analysis

All the data were expressed as the mean ± SD. The Chi-square and Fisher's exact tests were used where appropriate to analyze categorical data, with P<0.05 taken to indicate statistically significant differences.

## RESULTS

Demographics: Of the 2216 patients with normal renal function, 1630 (73.5%) were males and 586 (26.5%) were females with a M:F ratio of 2.7:1. The mean age was 7.1±3.8 years with a range of three months to 15 years. Age distribution showed 62 (2.8%) in the age group ≤ 1 year, 387 (17.5%) 1-3 years, 393 (17.7%) in 3-5 years, 873 (39.4%) in 5-10 years, 501 (22.6%) in 10–15 years.

In the 402 patients with renal failure, the mean age was 6.9±3.4 years with a M: F ratio of 2.0:1. However, within this group, anuric patients had a low mean age of 4.3±2.9 years with a range of nine months to 14 years.

Of the 2216 patients presenting with normal renal function 54% were from an urban area and 46% from a rural area. This was in contrast to the renal failure group where the majority (264, 66%) were from rural areas with a mean travel distance of 160±45 km and mean travel time of 14±6h. Past history of the patients revealed recurrent diarrhea in 23% and history of stone disease in 21%. Patients presenting with renal failure also gave history of previous stone surgery in 78 (22%) and positive family history of stone disease in 241 (60%). The majority of the patients (89%) were from the low income group at Rs. 2000/month ($35), 10.75% from the middle class and 0.25% from the upper middle or rich classes.

Clinical Presentation: Common clinical signs and symptoms are given in Table 2. In the normal renal function group, vague abdominal pain was found in 32% of the children ≤ 5 years of age as compared to 18% in those of ≥ 10 years. Of the 402 presenting with renal failure, 105 (26%) were anuric and 297 (74%) nonanuric. The mean duration of anuria was 4.3±2.3 days with a range 1-10 days. In the nonanuric group the mean duration of various symptoms was 250 days with a range of 365 to 2920 days.

**Table 2:** Clinical presentation in pediatric stone formers

	Normal renal function n = 2216		Renal failure n = 402	
		
	No.	%	No.	%
Fever	778	35.1	228	56.7
Flank pain	778	35.1	222	55.2
Vague abdominal pain	817	36.9	51	12.7
Hematuria	685	30.9	65	16.2
Nausea, vomiting	501	22.6	154	38.3
Dysuria/frequency	685	30.9	144	35.8
Shortness of breath	-	-	153	38.0
Oligo-anuric	-	-	105	26.1
Swelling	-	-	48	11.9

Hematology and Renal Function Biochemistry: In patients presenting with normal renal function the mean hemoglobin (Hb) was 9.5 g/dl (range 7.5-15 g/dl) and 20% of the patients were anemic (Hb>10 g/dl). Whereas in renal failure patients’ mean Hb was 7.6±2.4 g/dl (range 4.0-13.5) and 69% of the patients were anemic (Hb < 10 g/dl), leucocytosis >16000/cm was found in 32%. At presentation mean serum creatinine was 8.0±3.8 mg/dl, urea 203±74 mg/dl, sodium 134±7.6 mEq/L, potassium 5.1±1.4 mEq/L and bicarbonate 11± 5.2 mEq/L.

Anthropometric and dietary analysis: Anthropometric data in 2372 revealed growth deficit and malnutrition in our patient population. Z-score >-1 (in the normal range) for height was observed in 775 (35%). The remaining 65% were deficient, Z -1 to −2, 598 (27%), Z -2 to −3, 465 (21%) and 378 (17%) had Z <-3, being severely deficient. Z scores for weight showed normal range Z > -1 in 531 (24%) and the rest were deficient. Z -1 to −2 in 797 (36%), Z -2 to −3 in 686 (31%) and severely deficient in 202 (9%).

Food frequency questionnaire and dietary recall in 2176 patients revealed that the standard diet taken by these children was low in protein (74%), potassium (43%), fiber (48%), calcium (55%), with low intake of fluids in 55% and high consumption of oxalate in 57%, sodium in 39%, purine in 42% and refined sugar in 41%. More than half (60%) of the population was taking less than the recommended calories.

Urinary analysis: Urine analysis was undertaken in 2422. The mean urinary pH was 5.95±0.77. Fifty-five per cent had pH ≤ 5.5, 28% between 6-7 and 17% >7.0. The mean specific gravity was 1.018±0.06 with 48% having specific gravity of more than 1.020. Nitrite was positive in 33% and protein in 42%. Significant RBCs > 5 HPF were detected in 36%, WBCs > 10 HPF in 32% and crystals were seen in 54% of the urines. Crystal types were calcium oxalate in 45%, uric acid in 20%, ammonium hydrogen urate 5%, amorphous urates in 10% and struvite 7%. Xanthine crystals were seen in eight and cystine in eight patients.

Preoperative urine cultures: Overall cultures were positive in 1208 (46%) of the patients. Cultures were positive in 963 (43.5%) patients with normal renal function where common organisms isolated were *E. coli* in 38%, proteus species 5%, enterobacter 7% and Klebsiella in 7%. In the renal failure group, cultures were positive in 245 (61%). Organisms isolated were *E. coli* in 38%, Klebsiella 15%, Pseudomonas 11%, enterobacter 8% and proteus in 8%. The rest of the positive had mixed bacterial growth and other organisms. Blood cultures were undertaken in 117 of these patients with sepsis. Organisms were isolated in 25, 10 patients had *E. coli*, six pseudomonas, six staph aureus coagulase and three had Klebsiella.

Radiology and Imaging: Ultrasound (USG) KUB examination was done in all 2618 patients, intravenous urogram (IVU) was performed in 1133 patients with normal renal function, non-enhanced spiral CT scan of urinary tract in 39 cases and contrast enhanced spiral CT Scan in three cases.

Distribution of stones in the urinary tract by imaging is given in Table 3. The majority of the patients with normal renal function had unilateral calculi as compared to 14% in the renal failure group. Conversely, the majority in the latter group had bilateral calculi. Stones at multiple sites were also higher in the renal failure group. Staghorn calculi were present in 88 (22%) of the patients.

**Table 3:** Distribution of calculi in the urinary tract

	Normal renal failure n=2216 (%)	Renal failure n=402 (%)
Unilateral	1431 (65)	57 (14)
Upper tract	1028 (51)	52 (13)
Bladder	303 (14)	5 (1)
Bilateral	466 (21)	243 (60)
Renal	442 (20)	210 (52)
Ureteric	20 (0.9)	15 (3.5)
Stone at multiple sites	319 (14)	102 (26)
Upper tract	172 (7.5)	42 (11)
Upper Tract + Bladder	147 (6.5)	60 (15)
Total bladder	450 (20)	65 (16)

Measurement of stone size in the upper tract (size of largest stone where multiple stones were present) showed that the mean size was 1.6±0.8 cm with a range of 0.2-6.0 cm. Of the 2618 patients evaluated, 38% had renal stones larger than 1.5 cm. The mean size of bladder stones was 2.1±1.2 cm with a range of 0.5-6.5 cm.

Ultrasound findings were positive in 3234 renal units in the normal renal function group. Hydronephrosis was severe in 7%, moderate in 31% and mild in 25%. Cortical thickness was normal in 43%, mild reduction in 22%, moderate in 25% and severe reduction in 9% was observed. Congenital urological anamolies were found in 48 (2.2%) patients.

Of the 402 in the renal failure group, 797 (99%) renal units showed positive findings while five renal units were absent. Size of the kidney was small in 161 (20%) renal units. Within this group of renal failure patients, 105 who were anuric, 93% of the renal units showed varying degree of hydronephrosis. Size of the kidney was normal in 75% and 84% of the renal units had normal or only mildly reduced cortical thickness while 80% had normal renal parenchymal echogenecity. In contrast, non-anurics of the renal failure group had normal sized kidneys in 67% and small in 24%. Cortex was moderately to severely reduced in 41% and there was markedly increased echogenecity in 57%. In all 17 (4%) of these patients had congenital urological anomalies.

Blood Chemistry: Blood parameters were undertaken in 2391 patients. The majority of the patients had blood chemistry parameters within the normal range. The mean blood urea was 24.16 ± 8.3, creatinine 0.45±0.41, uric acid 3.99±2.1, calcium 10.25±4.9, phosphorus 5.7±2.25, magnesium 3.02±1.13, sodium 139.17±11.9, potassium 4.63±0.73, chloride 106.9±13.6, bicarbonate 21.85±4.1 and albumin 4.35±0.35. Borderline hypercalcemia, hyperuricemia and hyperphosphatemia was noted in 2%, 3% and 4% respectively.

Twenty-four hours urine study: Metabolic analysis was possible in 1892 patients. The mean values are given in Table 4. Significant findings were hypovolumia, hyperoxaluria and hypocitraturia. Risk factors were analyzed by stone type with increased and reduced levels marked by ↑ or ↓ with percentages in brackets [Table 4]. Hypovolumia (*P*=0.0001), hyperuricosuria (*P*=0.0001), hyperphosphaturia (*P*=0.009) and hyponatriuria (*P*=0.009) were significantly high in the ammonium hydrogen urate (AHU) group as compared to the calcium oxalate (CaOx) group while hypercalciuria (*P*=0.001) and hypernatriuria (*P*=0.001) were significantly high in the CaOx group as compared to the AHU group.

**Table 4:** Metabolic risk factors in 24h urine of pediatric stone formers

n= 1892 Parameter	Mean ± SD	Overall n = 1892	AHU stone formers n = 174	CaOx stone formers n = 264
Urine volume	975.8 ± 756	↓31%	↓49%	↓18%
Uric acid	14.0 ± 23.0	↑26%	↑45%	↑17%
Calcium	4.1 ± 9.6	↑26%↓20%	↑14%↓29%	↑30%↓13%
Phosphorus	14.4 ± 25.2	↑19%↓22%	↑29%↓20	↑12%↓16%
Sodium	65.5 ± 36.0	↑12%↓38%	↑5%↓54%	↑19%↓21%
Potassium	15.9 ± 10.3	↓47%	↓40%	↓40%
Magnesium	3.3 ± 12.6	↓11%	↓17%	↓9%
Ammonium	29.7 ± 24.6	↑11%	↑13%	↑15%
Protein	214 ± 322.9	↑39%	↑41%	↑37%
Oxalate	1.90 ± 6.2	↑43%	↑60%	↑36%
Citrate	3.58 ± 13.9	↓87%	↓83%	↓90%
PH	6.07 ± 2.4	↓50% ↑9%	↑15%↓48%	↑8%↓49%

**Figure 2:**
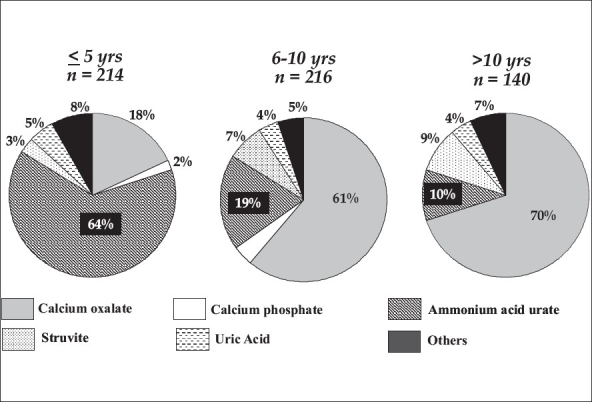
Distribution of pure renal stones in different age groups

Stone Analysis:Of the 2039 stones analyzed, 726 (36%) were composed purely of a single compound salt and 64% were mixed stones. Overall frequency of compounds in stones was AHU in 58%, calcium oxalate monohydrate (COM) in 63%, calcium oxalate dihydrate (COD) 20%, uric acid (UA) 6%, calcium phosphate apatite (CAP) 12% and struvite in 8%. Other stones in the kidney were cystine 1%, xanthine 1%, brushite 0.3%, protein 0.8% and 2,8 dihydroxy adenine 2, Newberyite 2 and whitlokite 4. Separate core and surface analysis was possible in 1274 renal and 198 bladder stones. Frequency of compounds in core of renal stones were AHU 64%, CaOx 41%, UA 3%, CAP 9% and struvite 5%. In the surface analysis, AHU reduced to 30% and CaOx increased to 72% while others were similar. Frequency of compounds in core of bladder stones was AHU 61%, CaOx 35%, UA 10%, CAP 7% and struvite 6%. Surface analysis again showed low AHU (37%), high CaOx (52%) and increased UA (16%), CAP (12%) and struvite (15%). Stratification of pure stones by age groups, A (≤5 years), B (6-10 years) and C (<10 years), showed differences in pattern of stones types. Group A, AHU 64%, CaOx 18%, Group B, AHU 19% and CaOx 61% while Group C, AHU 10% and CaOx 70% [Figure 2].

### Management

Definitive management was undertaken after treatment of UTI with culture-specific antibiotics. In 402 patients with renal failure, initial management included hemodialysis in 180 (45%), peritoneal dialysis in 60 (15%), PCN in 104 (26%), JJ stenting in 45 (11%), while two patients reported so late that they died during resuscitation. Definitive surgical management included open surgery and minimally invasive techniques. Of the 2485 patients treated, open surgery was undertaken in 721 (29%), PCNL in 679 (27.5%), ESWL in 323 (13%), cystolithoclast in 475 (19%) and URS in 287 (15.5%). Following stone clearance patients were given dietary advice and medication according to risk factors and stone type. Hydration, Lemonade and polycitra at 3-4 mEq/day was given to all patients except the struvite stone formers. Polycitra was homemade and given free to all patients. Risk factors in stone formers, especially those with CaOx and AHU were treated according to the following protocol.

Hyperoxalurics: Oxalate levels of <2 mg/kg/24h were advised dietary restriction of oxalate-rich foods, normal recommended calcium intake and those with > 2 mg/kg/24h were given pyridoxine 25–50 mg/day according to age along with dietary restrictions.

Hyperuricosurics: Dietary restriction of purine-rich foods and allupurinol in a dose of 10 mg/kg/day.

Hypercalciurics: Dietary restriction of excessive calcium and sodium. Thiazides at 1mg/kg/day especially with calcium renal leak.

Hypocalciurics: Milk supplements and restriction of oxalate-rich foods.

Cystine stone formers: They were advised dietary protein and sodium restriction and were given polycitra at 3-4mEq/kg/day to maintain pH > 7.5. Patients were advised to keep a close check of urine pH in every urine void with litmus paper provided to them.

Calcium phosphate stone formers: They were advised dietary restriction of phosphate-rich foods and calcium restriction and increase of fiber in their diet.

Uric acid stone formers: Patients were advised alkalization with 3-4 mEq/kg/day of polycitra to keep a urinary pH of ≥ 6.5, allupurinol 10 mg/kg/day and purine restriction where indicated.

Xanthine and 2–8 dihydroxyadenine stone formers: They were advised hydration and alkalization with polycitra and regular check of urinary pH by litmus paper.

Struvite stones: Patients with struvite stones were managed by complete stone removal including fragments. Where indicated anatomical abnormalities were corrected and long-term culture-specific antibiotics were given usually for six weeks and in some cases up to three months.

Follow-up metabolic analysis was undertaken in 1575 patients. Overall hypovolumia reduced from 31% to 5% (*P* <0.0001), hyperuricosuria from 26% to 6% (*P* < 0.0001), hypocalciuria from 20% to 5% (*P* < 0.0001), hypercalciuria from 26% to 5% (*P* <0.0001), hyperoxaluria from 43% to 15% (*P* <0.0001), hyponatriuria from 38% to 11% (*P* <0.0001), hypokaluria from 47% to 8% (*P* <0.0001) and hypocitraturia from 87% to 10% (*P* <0.0001). In 2216 patients with normal renal function recurrence was noted in 25 (1.15%) in the period of follow-up. Of these, eight had cystine stones, three xanthine, eight struvite, one uric acid and five patients had CaOx stones.

## DISCUSSION

Etiology of pediatric urolithiasis remains largely unknown in the developing world.[8,9] Western studies show anatomical abnormalities, infection and metabolic causes in up to 90% of the cases.[10] This emphasizes the need to enhance efforts to identify the risk factors and to elucidate the etiology and pathogenesis of stone disease in our region, especially in children since they are considered a high-risk group.[11] This necessitates a rational and practical evaluation protocol for risk identification and management. Several protocols have been suggested[12,13] which emphasize an initial basic evaluation and then an extensive workup depending on the stone type to determine urinary risk factors.

Evaluation protocol in our region requires a modified approach as 15% of the patients present with renal failure. We have therefore divided our evaluation into primary, initial management in patients with renal failure and detailed evaluation including history and examination, further imaging, stone analysis and metabolic studies [Figure 1]. Dietary and metabolic analysis is undertaken early to avoid repeated visits and to facilitate our patients who come long distances from rural and periurban areas.

Clinical signs and symptoms were nonspecific in both groups of patients with or without renal failure eluding diagnosis of stone disease. However, history of stone disease in a quarter of patients warranted a high index of suspicion. On this backdrop of vague symptoms, ultrasound examination of the abdomen was the mainstay of diagnosis and should be considered as an integral part of the extended physical examination. USG not only helped in the early diagnosis but also provided assessment pertaining to site and size of stones, especially non-opaque or faintly opaque stones. This was important when 40% of the children below five years harbored AHU stones which are usually radiolucent or faintly opaque. USG has several advantages over IUV. Firstly, in I, preparation is difficult, duration of examination is long and at times gives incomplete information and is not recommended in renal failure patients. Secondly, with USG repeated examinations on subsequent visits allows assessment of changing conditions i.e. stone position, hydronephrosis. However, IVU was important in patients who needed evaluation for associated anatomical details and for decision of PCNL or ESWL as the anatomy is well delineated. Non-enhanced spiral CT is an emerging imaging option particularly in cases of small, non-opaque stones in the lower half of the ureter, in presence of gas shadows and in patients where other abdominal pathology needs to be excluded. Contract enhanced spiral CT (CTU) is useful in complex cases, where other imagining modalities are not helpful.

Routine urine analysis by dipstick and microscopy is of value where nitrite positivity and pus cells were helpful findings for further culture tests. Presence of stone-specific crystals e.g. struvite, cystine, xanthine and AHU identify stone type. Large stone burden in half of the patients with upper and lower tract stones together with neglect predisposing them to infection. High rate of urine culture positivity is comparable to others,[13] however, of interest is the fact that urea-splitting organism were found in <15% of cases with major organism being *E. coli*. Moreover, *E. coli* was also predominant in patients with sepsis. It appears that UTI in our patients is secondary to stones and obstruction caused by stasis and delay in treatment. Low frequency of struvite stones (5-12%) is also reported by others in our region[14,15] which contrasts with the west where struvite constitutes 15-20%, frequently in association with anatomical abnormalities.[9,13]

Anthropometric findings were indicative of malnutrition as the majority of the patients were deficit in height and weight by Z scores and the majority were taking less than recommended calories.[6,7] The diet of these children was lithogenic as shown by high intake of oxalate and purine-rich foods and low consumption of protein, calcium, potassium and fiber. Most importantly about half of the children have fluid intake less than normally recommended.

Collection of 24h urine is a challenge, especially in younger children yet its analysis identified risk factors in the majority of the patients. Hypocitraturia was the commonest finding as reported by others in the region.[16] This is mainly due to low citrate in diet as shown by low potassium intake in our dietary analysis. Hyperoxaluria was found in nearly half of the patients probably due to diet rich in oxalate and low calcium intake secondary to a diet poor in milk and dairy products. Hyperuricosuria in a quarter of all patients and 50% in AHU stone formers is probably due to dietary factors as the diet was rich in wheat germ and legumes. However, other factors related to metabolic abnormalities cannot be excluded.[17] The other important finding was low urinary volume in a third of our patients. Inadequate consumption of fluids is in half of the patients is reflected by low 24 hour urinary volumes and concentrated urine in stat samples with specific gravity of > 1.020 in nearly half of the patients. These findings, together with hyponatriuria in 50% of the children and history of diarrhea in a quarter suggest that many children were dehydrated. In calcium oxalate stone formers hypocitraturia, hyperoxaluria and disturbances in calcium excretion were important findings similar to other series.[18] The AHU stone formers showed increased excretion of uric acid with high ammonium and low sodium and potassium as shown by others.[19]

Stone composition provided key information where core and surface analysis was possible. The core was composed of AHU in 34% of the pure renal stones. This increased to 66% in children less than five years of age. In contrast, core was composed of CaOx in 70% of children more than 10 years of age. The pattern of stone composition in older children was similar to that reported from developed countries where CaOx predominates.[9,13] However, AHU stones in the urinary tract reported from are region [14,15] are rare in developed countries.[9] The presence of AHU in 39% of the core of bladder calculi and equal distribution of CaOx is similar to other reported series.[15] The fact that AHU stones were found in the highest number in children below five years of age identifies and reemphasizes that malnutrition, diarrheal disease and dehydration as found in our cases is implicated in AHU formation as observed by others.[7,19]

The hallmark of stone management is complete stone removal and prevention of recurrence. In our patients it is equally important to treat infections due to stones and to preserve renal function as many patients present late with renal failure. This requires a multidisciplinary approach, all under one roof comprising intensivist, radiologist, pathologist, nephrologist, anesthesiologist and urologist. Such an approach was beneficial, especially in the oligo-anuric group who presented with acutely obstructed kidneys with normal cortex. The majority of these cases showed complete recovery of renal function. Although the majority of the patients were managed by minimally invasive techniques, a quarter needed open surgery due to large stone burden, impacted stones and compromised kidneys. The value of extensive protocol in these children who are at high risk can be appreciated from the finding that we were able to identify the risk factors in 90% of the patients. Dietary and pharmacological interventions enabled us to reduce risk factors in 2/3 of our patients. Low recurrence rates observed in our series as compared to others of up to 50%, where no medical intervention or follow-up was undertaken,[20] suggest the importance of identification of risk factors and their correction. Furthermore abundance of AHU stones may also contribute to low recurrence since thus far we have not observed recurrence in AHU stone formers. Our policy of giving polycitra together with hydration as proposed by others[21] helped reduce risk factors, especially in our major stone types, CaOx and AHU. Effects of citrate on CaOx crystallization and aggregation are well established and correction of sodium and potassium in urine may reduce availability of ammonium ions to couple with urates to promote AHU formation.[18,19] Although this extensive protocol identified risk factors in our patient population further research is needed to identify etiology and introduce preventive measures by public education.

## CONCLUSION

Pediatric stone formers present with vague urological symptoms where previous history gives an index of suspicion. Ultrasound is diagnostic in the majority of the cases and gives pertinent information on site, size of stone and anatomy of the renal units. On the backdrop of malnutrition, chronic dehydration and a diet poor in proteins and rich in oxalates, lead to formation of AHU stones which constitute up to 64% of stones in children <5 years of age, who are most likely to suffer from these risk factors. Calcium oxalate lithogenesis in these children is a byproduct as a result of AHU nidus while it is the main disease in older children where hyperoxaluria, hypocalciuria and dietary deficiency of citrates and dehydration may well be the main risk factors. Both AHU and CaOx stones thus seem preventable where dietary modifications and diarrheal disease may play a pivotal role. In our experience true assessment of risk involves a trio of evaluation to include diet, 24h urinary analysis and composition of stones. These can identify the risks in the majority of the children with stones. Dietary and medical management can help reduce risk factors by two-thirds resulting in recurrence rates of about 1%. A systematic evaluation protocol in the stone clinic will help in management strategies and is practical and affordable from the developing world's perspective.
